# Development of Efficient, Reproducible and Stable *Agrobacterium*-Mediated Genetic Transformation of Five Potato Cultivars

**DOI:** 10.17113/ftb.58.01.20.6187

**Published:** 2020-03

**Authors:** Allah Bakhsh

**Affiliations:** Department of Agricultural Genetic Engineering, Faculty of Agricultural Sciences and Technologies, Nigde Omer Halisdemir University, 51240 Nigde, Turkey

**Keywords:** potato genetic transformation, genotypes, plant growth regulators, cost-effective protocol

## Abstract

The developments in transformation technology have enabled the scientists to incorporate, mutate or substitute gene(s) leading to a particular trait; advancing it to a point where only few technical limitations remain. Genotype dependency and explant types are important factors affecting transformation efficiency in potato. In the present study, a rapid, reproducible and stable *Agrobacterium*-mediated transformation procedure in potato was developed by a combination of different plant growth regulators. Leaf discs and internodal explants of five cultivars of potato, *i.e.* Lady Olympia, Granola, Agria, Désirée and Innovator were infected with *Agrobacterium tumefaciens* strain LBA4404 containing pBIN19 expression vector with β-glucuronidase *gusA* gene under the control of 35S CaMV promoter. Kanamycin was used as plant selectable marker for screening of primary transformants at concentration of 100 mg/L. Both explants responded positively; internode being more suitable explant for better transformation efficiency. Based on GUS histochemical assay, the transformation efficiency was 22, 20, 18.6, 15 and 10% using the internodal explant, and 15, 12, 17, 8 and 6% using leaf discs as explant in Lady Olympia, Granola, Agria, Désirée and Innovator respectively. Furthermore, PCR assays confirmed the presence of *gusA* and *nptII* genes in regenerated plants. The molecular analysis in succeeding progeny showed proper integration and expression of both genes. The results suggest Lady Olympia as the best cultivar for future transformation procedures. Overall, the short duration, rapidity and reproducibility makes this protocol suitable for wider application of transgenic potato plants.

## INTRODUCTION

Potato (*Solanum tuberosum* L.) is a primary non-cereal food crop and ranks 4th in the world because of its high productivity, following corn (*Zea mays* L.), rice (*Oryza sativa* L.), and wheat (*Triticum aestivum* L.) in terms of yield, acreage and value (*1*). Potato is considered one of auspicious crop to overcome the challenges of poverty and hunger worldwide (*2*). Turkey is an important potato producing country globally. Potato accounts for 3% of the gross national agricultural product, 3.1% in 27 EU countries, therefore has a significant impact on Turkish economy (*3*).

Potato is affected by viral, bacterial and fungal diseases, and nematodes (*4*). The crop is affected by pathogens at different stages of growth, from the preharvest to the postharvest (*5*). Using modern technologies, the researchers have incorporated different traits related to biotic and abiotic stress tolerance in potato. These technologies transcend traditional plant breeding methods by allowing the rapid and predictable gene transfer across the species boundaries. None of the commercial transgenic potatoes is being planted until the completion of regulatory and safety process and later on commercial release of Innate^TM^ 1.0 potatoes in Atlantic, Ranger Russet, and Russet Burbank cultivars with low acrylamide potential and black spot bruise resistance traits by J.R. Simplot Company (*6*).

*Agrobacterium*-mediated genetic transformation is widely used for the introduction of foreign gene(s) into dicots (*7*). There are several studies that report the successful incorporation of foreign gene(s) in potato using *Agrobacterium*-mediated transformation (*8*-*10*). Using two-stage transformation protocol with leaf explants, Cingel *et al.* (*9*) successfully reported high shoot regeneration efficiency (84-89% for Dragacevka and 60-68% for Jelica cultivar compared to 76-86% for Desiree) by combining procedures of Webb and Wenzler. However, from a broader perspective, these published procedures face limitations of lower transformation frequency, genotype dependency or somaclonal variations mainly due to prolonged tissue culture conditions, thus resulting in lower yield of stable transgenic plants (*11*).

Development of an efficient and reproducible plant transformation and regeneration protocol is a prerequisite for genetic transformation studies of plants (*12*). The successful plant transformation requires a proper DNA delivery system, a plant regeneration system, and a selection system to recognize the transgenic cells. As evident from scientific literature, an efficient and reproducible transformation protocol in potato is still lacking; therefore, by combining different already reported protocols with some modifications, the present study attempts to optimize a cost-effective and reproducible transformation protocol for five different potato cultivars, *i.e.* Lady Olympia, Agria, Granola, Désirée and Innovator.

## MATERIALS AND METHODS

### Potato cultivars

The present study uses five commercial cultivars (Lady Olympia, Agria, Granola, Désirée and Innovator) of potato growing zone in Turkey for genetic transformation. These cultivars have been selected on the basis of their agronomic characteristics and good yield potential in Central Anatolian Region, Turkey.

### Establishment of shoot cultures

Tuber sprouts of the aforementioned potato cultivars were surface sterilized as described by Bakhsh *et al.* (*13*) for sweet potato. The *in vitro* shoot cultures of cultivars obtained from tuber sprouts were propagated monthly as nodal cultures by incubating on medium containing Murashige and Skoog (MS) (*14*) mineral salts, 3% sucrose and 8 g/L plant agar. The pH of the medium was adjusted to 5.7-5.8 prior to autoclaving. All chemicals (plant agar, MS salts and sucrose) used in this study were purchased from Duchefa Biochemie B.V. (Haarlem, the Netherlands) and Sigma-Aldrich Co., Merck (St. Louis, MO, USA). Cultures were grown under controlled conditions in a growth room with a 16/8 h light/dark photoperiod, 47 µmol/(m-^2^·s) irradiance at the culturing surface provided by 58 W fluorescent tubes and temperature (25±2) °C.

### Agrobacterium-mediated transformation

**Fig. 1:**
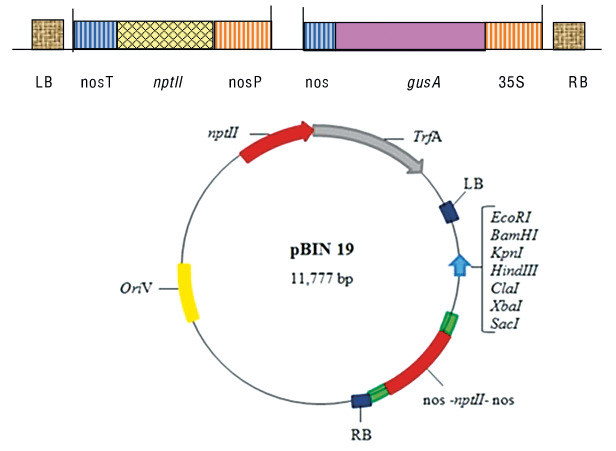
Description of pBIN19 binary vector containing *gusA* and neomycin phosphotransferase (*nptII*) driven by cauliflower mosaic virus (CaMV35S) promoter and nopaline synthase (NOS) terminator in between right and left border. The construct has *nptII* gene that encodes resistance to kanamycin, used as a plant selectable marker at *γ*=100 mg/L

*Agrobacterium tumefaciens* strain LBA4404 harbouring pBIN19 binary vector containing *gusA* (β-glucuronidase) gene under the control of 35S promoter was used (Fig. 1). The reporter gene (*gusA*) was interrupted by an intronic sequence to induce expression only from eukaryotic cells. The glycerol stocks were streaked on Luria-Bertani (LB) agar plates containing kanamycin and rifampicin (Sigma-Aldrich Chemicals Co., Merck, St Louis, MO, USA) each at concentration of 50 mg/L. One colony from a plate was inoculated in LB broth supplemented with appropriate concentrations of antibiotics. The bacterial culture was incubated in shaking incubator (ISF1-X; Adolf Kühner AG, Basel, Switzerland) at 28 °C overnight.

The explants were inoculated with *Agrobacterium* suspension (*A*_600 nm_=0.8) for 20 min with gentle shaking at 180 rpm in liquid medium without antibiotic followed by incubation on co-cultivation medium for approx. three days. Cocultivation medium contained MS salts including vitamins, 3% sucrose, 7-8 g/L agar and 100 µM acetosyringone (Sigma-Aldrich Chemicals Co., Merck). The pH of the medium was adjusted to 5.8. After cocultivation, the explants were blotted dry on a filter paper and cultured with three different hormonal combinations on three regeneration selection media (RSM) as three concurrent experiments, *i.e.* RSM1 consisting of MS supplemented with *trans*-zeatin 2 mg/L+naphthaleneacetic acid (NAA) 0.2 mg/L, RSM2 with benzyladenine (BA) 2 mg/L+NAA 0.2 mg/L+gibberellic acid (GA3) 0.1 mg/L, and RMS3 with BA 2 mg/L+NAA 0.2 mg/L+*trans*-zeatin 2 mg/L+GA3 0.1 mg/L. Regeneration selection media were used for both callus induction and shoot induction. The plant growth regulators used were purchased from Duchefa Biochemie B.V. Duocid 300 mg/L (Pfizer, Istanbul, Turkey) and kanamycin 100 mg/L were also added to each RSM to eliminate excessive growth of *Agrobacterium* and for selection of primary transformants, respectively.

The *in vitro* cultured shoots reaching a length of 2–3 cm were excised and transferred to large magenta boxes (107x94x96 mm) containing MS medium with kanamycin 100 mg/L to continue selection pressure. The plantlets with well-established roots were shifted to soil comprising peat and perlite mix (3:1) in a greenhouse to obtain tubers for further studies.

### GUS histochemical assay

GUS histochemical assays were performed to test the functionality of *gusA* gene at different steps of transformation. To accomplish the task, GUS buffer containing 10 mg/L X-Gluc, 10 mM EDTA, 100 mM NaH_2_PO_4_, 0.1% Triton X-100 and 50% methanol (Sigma-Aldrich Chemicals Co., Merck) was prepared. The pH of the buffer was maintained at 8. The resistant calli, regenerated shoots and plantlets were incubated in this buffer overnight at 37 °C. Furthermore, samples were destained by adding 70% ethanol to 2-mL Eppendorf tubes. The transformation efficiency in cultivars was calculated based on the GUS activity.

### PCR analysis

The putative transgenic plants belonging to different cultivars were analyzed by PCR assays to confirm the presence of *gusA* and *nptII* genes with introduced T-DNA. For this purpose, genome DNA was isolated using Nucleo Spin^®^ Plant II Kit (Macherey-Nagel GmbH & Co., Düren, Germany) and then PCR assays were carried out using gene-specific primer to amplify internal fragments of the aforementioned genes. PCR reaction was carried out in a total reaction volume of 20 μL containing 1× reaction buffer, 10 ng DNA template, 1.5 mM MgCl_2_, 0.1 mM dNTPs mix, 0.5 µM each primer and one unit Taq DNA polymerase (Thermo Scientific Inc. Waltham, MA, USA). Furthermore, the reaction was carried out using initial denaturation at 94 °C for 4 min followed by 35 cycles of denaturation at 94 °C for 30 s, annealing at 55 °C (*gusA* and *nptII*) and 65 °C (*chvA*) for 30 s, extension at 72 °C for 30 s, followed by a final extension at 72 °C for 7 min. In all PCR reactions, pBIN19 plasmid was used as positive control whereas the DNA isolated from untransformed plants was used as negative control. Table S1 contains information about primer sequences, annealing temperature and product size.

Nester (*15*) reported that *Agrobacterium* infection of plant host depends on activation of chromosomal virulence genes along with *vir* gene. Since c*hvA* gene has a role in the attachment of bacteria to the host cell, the primary transformants containing any *Agrobacterium* contamination (latency) were tested using gene-specific primers.

The transgenic potato plants positive for the introduced gene (based on PCR and GUS histochemical assays) were grown in a greenhouse, tubers were harvested and planted again to confirm the presence and expression of *gusA* in succeeding tuber progeny. All statistical analyses were performed with Statistix v. 8.1 software (*16*). Significance of variance was determined after the one-way ANOVA (p<0.05) followed by least significance difference (LSD) test.

## RESULTS AND DISCUSSION

The scientific literature reports the use of *Agrobacterium*-mediated genetic transformation system for the introduction of gene(s) encoding traits of economic importance in crop plants (*17*, *18*). *Agrobacterium tumefaciens* strain LB4404 has been found more infective and efficient than other strains for transformation especially in dicots (*19*). In the present study, strain LBA4404 harbouring recombinant plasmid pBIN19 was used to optimize the transformation protocol in potato cultivars under study.

**Fig. S1:**
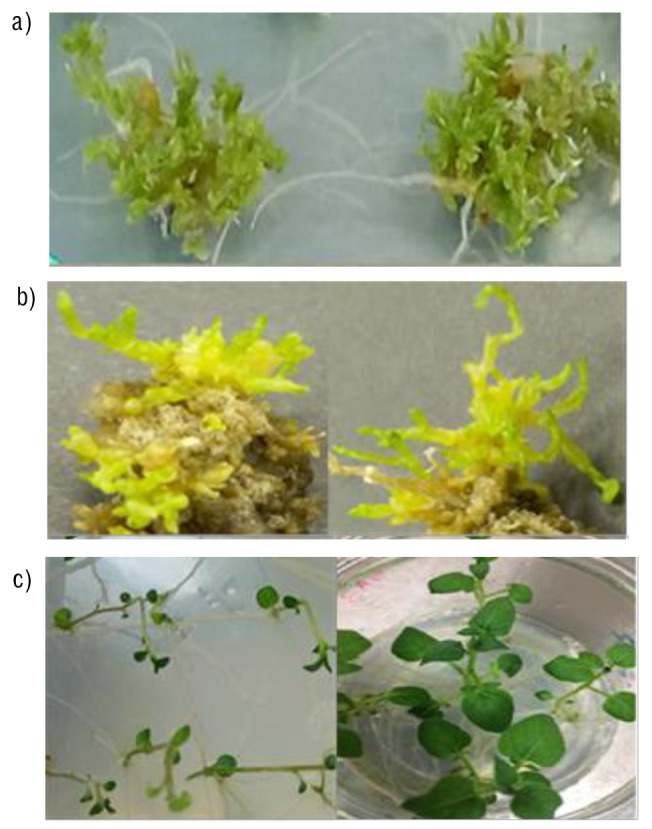
Different steps of genetic transformation of potato cultivars: a) regenerated calli from internodal explants of Lady Olympia cultivar, b) regenerated resistant calli from Agria cultivar, and c) putative transformed shoots on selection media with induced roots, ready for acclimatization

In optimization experiments, three different regeneration selection media (RSM) were used for leaf discs and internodal explants (Table 1 and Table 2). Fig. S1 shows different steps of genetic transformation. Leaf disc explants cultured on RSM3 showed good results compared to other two selection media, although the response of cultivars also varied. The maximum number of resistant calli was observed in Lady Olympia cultivar followed by Désirée, Agria, Granola and Innovator (Table 1). The average number of shoots per explant was high in Désirée, followed by Lady Olympia, Agria, Granola and Innovator. The response of internodal explants cultured on RSM3 was also more encouraging than on the other two selection media. The maximum percentage of resistant calli development was observed in Désirée, followed by Lady Olympia, Agria, Granola and Innovator, whereas average number of shoots per explant was high in Désirée and Agria, followed by Lady Olympia, Granola and Innovator (Table 2). Both explants showed encouraging response of callus induction and its further proliferation. The shoot regeneration worked better from internodes of certain cultivars, while for others it worked better from leaf discs. Shoot development using internodal and leaf explants of Lady Olympia and Désirée was not significantly different on RSM3; however, it was different when compared to other cultivars. Almost 100% rooting was observed in regenerated shoots. The well rooted regenerated plants were transferred to the greenhouse and subjected to GUS histochemical assays at different stages.

**Table 1:** Study of the influence of different regeneration selection media (RSM) on the regeneration of potato cultivars using leaf discs as explant

Cultivar	RSM1			RSM2			RSM3		
*N*(explant)	Callus induction/%	*N*(shoot)*N*(explant)	*N*(explant)	Callus induction/%	*N*(shoot)*N*(explant)	*N*(explant)	Callus induction/%
Lady Olympia	300	70.0^a^	1.80^ab^	300	76.5^a^	3.55^a^	300	82.2^a^	4.75^ab^
Désirée	300	60.0^b^	2.20^a^	300	60.0^c^	2.50^a^	300	80.0^a^	5.50^a^
Agria	300	61.5^b^	1.25^ab^	300	66.6^b^	1.20^b^	300	70.5^b^	2.70^c^
Granola	300	42.2^c^	0.00^b^	300	44.0^d^	0.80^b^	300	45.6^c^	2.55^bc^
Innovator	300	30.1^d^	0.00^b^	300	32.5^e^	0.00^b^	300	32.0^d^	1.50^c^

Mean values followed by the same letter in superscript within a column are not significantly different according to LSD test at p=0.05

*γ(kanamycin)=100 mg/L and *γ*(duocid)=300 mg/L were added to each RSM medium for plant selection and to inhibit bacterial growth, respectively. RSM1=*trans*-zeatin 2 mg/mL+naphthaleneacetic acid (NAA) 0.2 mg/L, RSM2=benzylademine (BA) 2 mg/L+NAA 0.2 mg/L+gibberellic acid (GA3) 0.1 mg/L, RSM3=BA 2 mg/mL+NAA 0.2 mg/L+*trans*-zeatin 2 mg/mL+GA3 0.1 mg/L

**Table 2:** Study of the influence of different regeneration selection media (RSM) on the regeneration of potato cultivars using internodes as

Cultivar	RSM1			RSM2			RSM3		
*N*(explant)	Callus induction/%	*N*(shoot)*N*(explant)	*N*(explant)	Callus induction/%	*N*(shoot)*N*(explant)	*N*(explant)	Callus induction/%
Lady Olympia	300	63.0^b^	1.35^ab^	300	58.5^b^	1.80^ab^	300	79.5^a^	4.50^ab^
Désirée	300	60.0^c^	2.50^a^	300	70.0^a^	2.50^a^	300	80.0^a^	5.50^a^
Agria	300	75.2^a^	2.20^a^	300	71.5^a^	1.20^ab^	300	74.5^b^	5.50^a^
Granola	300	48.7^d^	0.45^b^	300	52.0^c^	0.00^b^	300	55.5^c^	3.00^bc^
Innovator	300	27.5^e^	0.00^b^	300	32.5^d^	0.00^b^	300	36.2^d^	2.50^c^

Mean values followed by the same letter in superscript within a column are not significantly different according to LSD test at p=0.05

*γ(kanamycin)=100 mg/L and *γ*(duocid)=300 mg/L were added to each RSM medium for plant selection and to inhibit bacterial growth, respectively. RSM1=*trans*-zeatin 2 mg/mL+naphthaleneacetic acid (NAA) 0.2 mg/L, RSM2=benzylademine (BA) 2 mg/L+NAA 0.2 mg/L+gibberellic acid (GA3) 0.1 mg/L, RSM3=BA 2 mg/mL+NAA 0.2 mg/L+*trans*-zeatin 2 mg/mL+GA3 0.1 mg/L

**Fig. 2:**
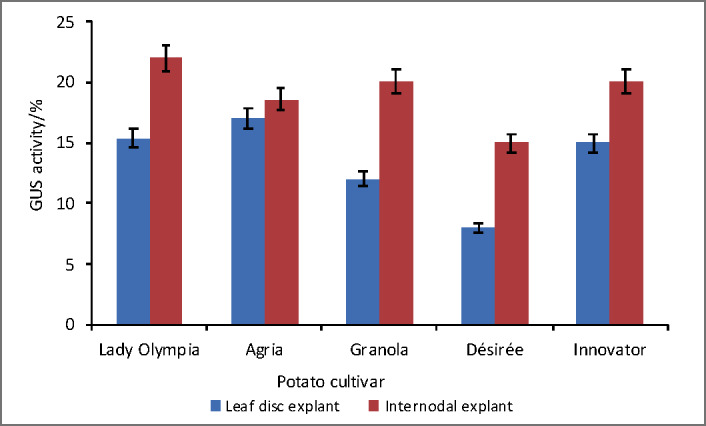
Transformation efficiency was recorded in putative transgenic shoots of cultivars Lady Olympia, Agria, Granola, Désirée and Innovator based on GUS histochemical assay using Leaf disc and Internodal explants

**Fig. S2:**
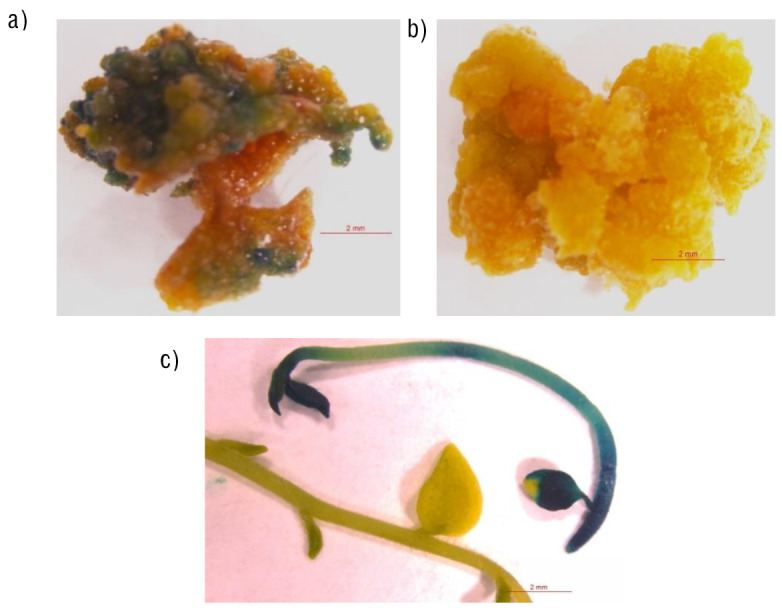
GUS histochemical assay used at various stages of potato transformation: a) resistant internodal explants on regeneration selection media, b) Non-transgenic callus calli, and c) GUS expression in *in vitro* transgenic plants at later stage

GUS histochemical analysis revealed the functionality of *gusA* gene in primary transformants at different stages (Fig. S2). Based on GUS assay results, higher transformation efficiency was recorded in Lady Olympia and Agria than in other cultivars in both explants. Encouraging and efficient transformation efficiency was recorded in all cultivars, although internodal explants proved to be more productive (Fig. 2). The combination of the *Agrobacterium* and the continuous selection pressure caused by kanamycin resulted in putatively transformed plants of each cultivar.

Previous studies by Banerjee *et al.* (*20*), Ducreux *et al.* (*21*) and De Block (*22*) have established organogenesis in potato using NAA, zeatin and GA3 combinations; however, the addition of BA and high concentration of zeatin in this study resulted in higher regeneration frequency that lead to increased number of shoots per explants than other regeneration selection media.

Genotype dependency in potato cultivars has also been observed when subjected to *Agrobacterium* transformation (*23*). Some of the cultivars are more amenable to transformation than others. Various factors, such as the type of vector and *Agrobacterium* strain, explant type, and varietal genetic background can affect the efficiency of *Agrobacterium*-mediated transformation in crops (*9*, *12*, *18*, *19*, *24*). The putative transgenic plants (210 in total number) of different cultivars were acclimatized to the greenhouse and confirmed by PCR assays. In this study, encouraging results were recorded from cultivars indicating the genotype independency to certain extent.

**Fig. 3:**
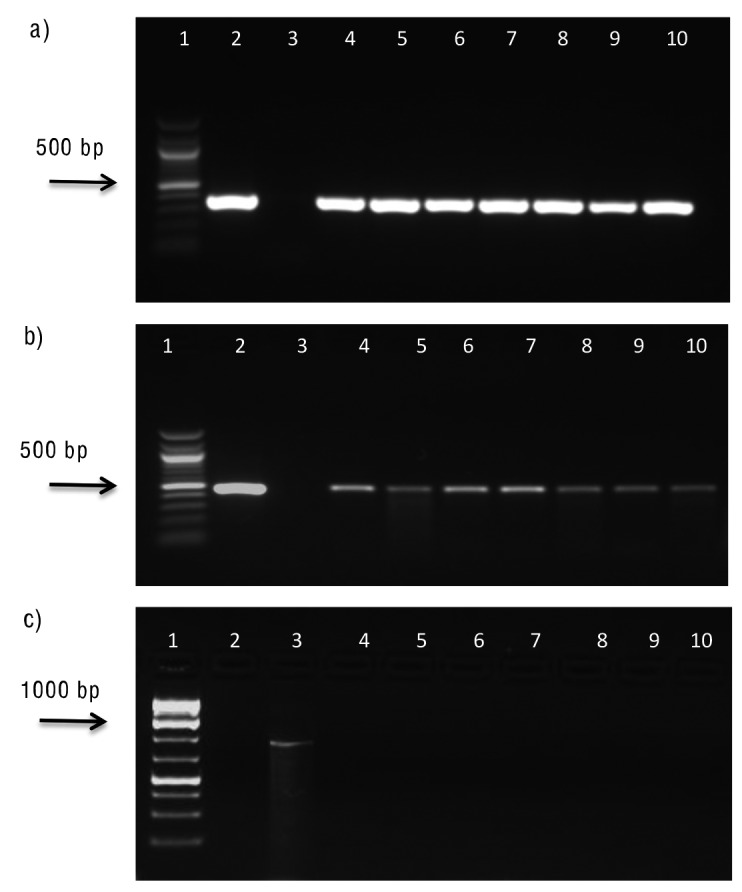
Molecular evaluation of primary transformants: a) PCR assay showed the amplification of internal fragment (362 bp) of *gusA*. Lane 1=100 bp plus ladder (Biolab), Lane 2=positive control plasmid, Lane 3=negative control, Lanes 4-10=representative putative transgenic plants of each cultivar; b) PCR assay showed the amplification of internal fragment (450 bp) of *nptII* gene. Lane 1=100 bp plus ladder (Biolab), Lane 2=positive control plasmid, Lane 3=negative control, Lanes 4-10=representative putative transgenic plants of each cultivar; and c) PCR results showed no amplification of *ChvA* gene in primary transformants indicating no any agrobacterium contamination. Lane 1=1 kb plus DNA ladder (Thermo Scientific), Lane 2=negative control, Lane 3=*Agrobacterium* chromosomal DNA as positive control, Lanes 4-10=primary transformants

The primary transformants showed amplification of required bands of 362 bp of *gusA* and 450 bp of *nptII* genes when subjected to PCR assays (Fig. 3a and Fig. 3b). In order to confirm the presence of any *Agrobacterium* contamination in primary transformants, PCR assays using *ChvA* gene (*Agrobacterium* chromosomal gene)-specific primers were also performed. Results revealed the absence of 890-bp amplicon, establishing the absence of any *Agrobacterium* contamination (Fig. 3c). *Agrobacterium* chromosomal genes are commonly used to test primary transformants (*15*, *25*, *26*).

**Fig. 4:**
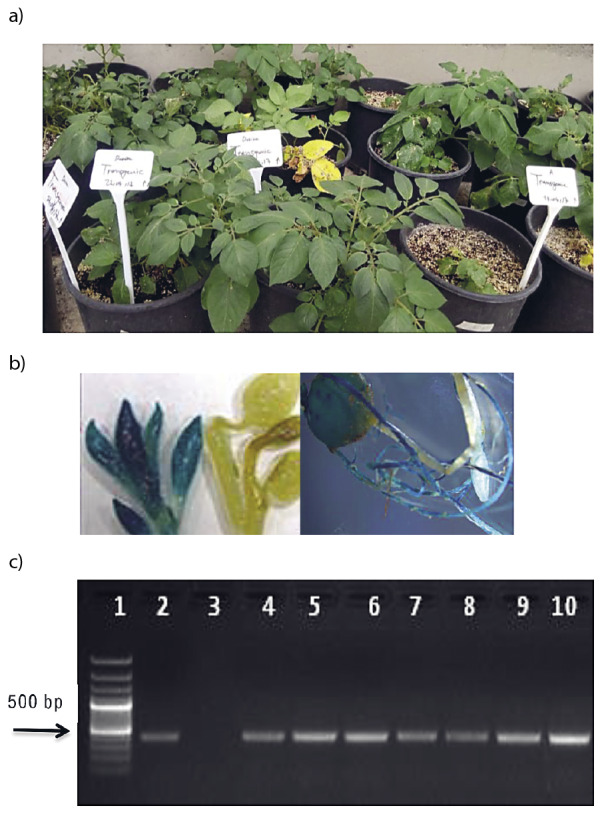
Evaluation of transgenic plants from first tuber generation: a) plant samples in the greenhouse, b) GUS histochemical assay showed the expression of *gusA* gene in transgenic plants, and c) PCR assay showing amplification of 450-bp internal fragment of *nptII* gene in transgenic plants. Lane 1=100 bp plus ladder (Thermo Scientific), Lane 2=positive control, plasmid DNA, Lane 3=negative control, Lanes 4-13=first tuber generation transgenic plants of Lady Olympia and Désirée cultivars

The harvested tubers from primary transformants were planted in the greenhouse (Fig. 4a) to confirm the expression of *gusA* gene in succeeding progeny by GUS histochemical assays (Fig. 4b). Besides that, PCR amplification of 450-bp partial fragment of *nptII* gene indicated the integration of T-DNA region in transgenic plants (Fig. 4c). First generation tubers were planted in the greenhouse to observe the introduced stability of introduced genes in subsequent progeny. GUS histochemical and PCR assays exhibited the gene transfer to subsequent progeny indicating the stability of the protocol.

The overall results showed that using proper hormonal combination in regeneration selection media can lead to increased regeneration frequency of potato cultivars. Using the above-mentioned protocol, insect- and herbicide-resistant potato lines expressing *cry1Ac* gene (*27*), *cp4-epsps* (*28*) and hairpin construct of insect metamorphosis-associated gene (*29*) were developed.

## CONCLUSION

The present study presents suitable hormonal composition of the regeneration media, type of explants and most suitable cultivar for *Agrobacterium*-mediated potato transformation. According to the results, the use of internode explants is recommended as a generally better procedure taking all aspects of transformation and regeneration into account. Additionally, Lady Olympia cultivar appears to be superior to others in terms of transformation efficiency. Previous studies report Désirée as a suitable cultivar for genetic manipulations; however, based on the results of regeneration and transformation, Lady Olympia can also be recommended for future transformation procedures.

## Figures and Tables

**Table S1:** Primers used in PCR studies to detect introduced gene(s) in primary potato transformants

Primer	Sequence (5’-3’)	Product size/bp	*t*(annealing)/°C
*npt-II*	F: TTGCTCCTGCCGAGAAAG R: GAAGGCGATAGAAGG CGA	450	55
*gusA*	F: CCCTTACGCTGAAGAGATGC R: GAGCGTCGCAGAACATTACA	362	55
*chvA*	F: CGAAACGCTGTTCGGCCTGTGG R: GTTCAGCAGGCCGGCATCCTGG	890	65
